# Effective Amelioration of Liver Fibrosis Through Lentiviral Vector Carrying *Toxoplasma gondii gra15_II_* in Murine Model

**DOI:** 10.3389/fimmu.2018.01572

**Published:** 2018-07-06

**Authors:** Lei Liu, Mengmeng Jin, Qing Tao, Li Yu, Jian Du, Cong Wang, Qingli Luo, Tian Xing, Yuanhong Xu, Jilong Shen, Deyong Chu

**Affiliations:** ^1^Department of Pathogen Biology and Provincial Laboratories of Pathogen Biology and Zoonoses, Anhui Medical University, Hefei, China; ^2^Key Laboratory of Oral Disease Research of Anhui Province, Stomatologic Hospital and College, Anhui Medical University, Hefei, China; ^3^Diagnostic Laboratory of the First Affiliated Hospital, Anhui Medical University, Hefei, China

**Keywords:** GRA15, lentiviral vector, schistosomiasis hepatic fibrosis, gene delivery, macrophage

## Abstract

Our previous investigations indicated that *in vitro* polarization of mouse macrophages by *Toxoplasma gondii* type _II_ strain dense granule protein 15 (GRA15_***II***_), one of the genotype-associated effectors of *T. gondii*, induced the phenotypes of classically activated macrophage (M1). Transfusion of the cells to mice may effectively alleviated hepatic fibrosis caused by schistosomiasis. The purpose of the study was to identify whether liver macrophages can be *in vivo* driven to M1 macrophages by lentiviral vector (LV) carrying GRA15_***II***_ gene (LV-*gra15_**II**_*) and to explore the potential mechanism by which the LV-*gra15_**II**_*-activated liver macrophage (LV-*gra15_**II**_*-M) ameliorates the hepatic fibrosis in schistosomiasis. The mice were treated with LV-*gra15_**II**_* by hydrodynamic injection *via* the tail vein followed by challenge of *Schistosoma japonicum* (*S. japonicum*). Our experiments showed that LV-*gra15_**II**_* was successfully delivered to liver macrophages and GRA15_II_ was persistently expressed in the macrophages of mice for at least 2 months. Furthermore, the LV-*gra15_**II**_* infected macrophages were polarized to M1 macrophages *in vivo*. Consequently, mice with schistosomiasis receiving LV-*gra15_**II**_* injection displayed a remarkable amelioration of liver granuloma formation and collagen deposition in association with downregulated expression of transforming growth factor-beta1, arginase 1 (Arg-1), α-smooth muscle actin, and an increased expression of matrix metalloproteinase 13 (MMP13). Simultaneously, no negative effects of liver function and vitality of mice were noted. The *in vitro* experiments indicated that the C-C motif chemokine ligand 2 and nitric oxide level were elevated in LV-*gra15_**II**_*-M cultural supernatants; hepatocyte growth factor expression was enhanced in LV-*gra15_**II**_*-M. In addition, LV-*gra15_**II**_*-M not only secreted MMP13, which greatly degraded type _I_ collagen, but also induced murine hepatic stellate cell (HSC) line (JS1) apoptosis in the co-culture system. Taken together, we identified for the first time that LV-*gra15_**II**_* may *in vivo* drive liver macrophages to M1 macrophage phenotypes, which helps for alteration of the liver fibrotic microenvironment with collagen dissolution, HSC deactivation, apoptosis and hepatocyte protection. Our study gives an insight into the use of gene delivery with parasite-derived immunomodulatory factor as a potential immune cell activating agent to re-equilibrate the other pathogen-induced immune response in some chronic diseases.

## Introduction

Liver fibrosis is caused by diverse etiologies and is a common pathological process which leads to end-stage liver diseases. Advanced liver fibrosis results in cirrhosis, portal hypertension, hepatocarcinoma, and liver failure (1). These serious disorders are associated with significant mortality risk and considerable financial burdens on health care systems worldwide. Liver transplantation, the only curative treatment for end-stage liver diseases, is limited by the shortage of available donors, the high cost of the procedure, and the life-long immune suppression to patient (2). In addition, cytotherapy for liver fibrosis has shown some adverse outcomes and unstable therapeutic effects, due to incomplete understanding of the cellular and molecular mechanisms (1, 2). Alternatively, gene therapy may be sufficient to overcome these problems. Lentiviral vector (LV) can transduce enough length of gene sequence into both replicating and quiescent cells without normal cellular functions *in vitro* or *in vivo* compromised. One of the key features of LV is the low anti-vector immunity inherent in host organisms, which is critical for the LV to avoid the rapid clearance of the vectors and the transgene-expressing cells by the host. The rarity of pre-exposure to lentiviruses in the hosts results in a general lack of pre-existing immunity against LV. So, LV is now used in the research of gene functions and for the gene therapy (3–6).

Liver fibrosis is a T helper cell type 2 (Th2)-dependent condition with numerous plastic and heterogeneous liver macrophages, such as classically activated macrophage (M1) and alternatively activated macrophage (M2), which adopt diverse phenotypes and functions during distinct phase of fibrosis (7). The two different macrophages activation statuses are tightly related with T helper cell type 1 (Th1) and Th2 lymphocyte polarizations. Th1 inflammatory cytokines, such as interferon (IFN)-γ, interleukin (IL)-1, and tumor necrosis factor-alpha (TNF-α), can induce M1 macrophage activation, which expresses Th1-polarizing cytokines and abundant inducible nitric oxide synthase (iNOS). In the M1 macrophage, iNOS actively competes with scarce arginase 1 (Arg-1) to oxidize l-arginine into nitric oxide (NO), which may inhibit the production of collagen, promote the apoptosis of hepatic stellate cell (HSC), and kill pathogens (7–10). In contrast, Th2 inflammatory cytokines, such as IL-4 and IL-13, can lead to M2 macrophage polarization, which produces Arg-1 and other factors involving in restriction of T-cell proliferation and activation. In the M2 macrophage, Arg-1 competitively uses l-arginine as a substrate to generate l-ornithine, which is converted to proline and polyamine. Proline is a crucial amino acid for the production of collagen and the development of fibrosis, whereas polyamine can give rise to fibroblast proliferation (7–9). Thus, M1 macrophages contribute to the amelioration of fibrosis (11, 12). In short, macrophages are central players in liver fibrosis, and they exert bidirectional roles in regulating matrix deposition and resolution (13–15). Thus hepatic macrophage has become a potential therapeutic target in treatment of liver fibrosis (16).

*Toxoplasma gondii* (*T. gondii)* is an obligatory intracellular parasite which is capable of infecting almost all warm-blooded animals, including humans (17). *T. gondii* releases some important effector molecules, such as dense granule protein 15 (GRA15) and rhoptry kinase 16 from secretory organelles into the host cell cytoplasm, which alter host cell transcription and activation through regulating host cell signaling pathways (18–24). Saeij, et al reported that *T. gondii* type II strain GRA15 (GRA15_II_) released into mouse macrophage cytosol during and after the parasite invasion to macrophage, can activate nuclear factor (NF)-κB, which brings about macrophage polarization toward a M1 phenotype, whereas *T. gondii* type I or III strain GRA15 (GRA15_I/ III_) has a negligible effect on NF-κB activation (22, 24).

Schistosome worm initially elicits a Th1-skewed/M1 macrophage-dominated response. After 4–6 weeks of infection, schistosome eggs trapped in tissues induce M2 macrophage-rich granulomas and Th2-biased immunity, which activates HSCs to synthesize collagens that help to confine toxic egg-derived antigens and protect surrounding parenchymal cells from damage (7). Normal granulomatous inflammation and M2 macrophages enable eggs to cross the intestinal wall into the lumen while accelerating wound healing, which prevents the enteric bacterial infection. Nevertheless, the excessive granuloma formation causes severe Th2-dependent liver fibrosis, which dramatically obstructs blood flow and consequently, leads to portal hypertension and even death in the chronically infected host. In contrast, if an exaggerated Th1 response develops against eggs, IFN-γ will activate M1 macrophages that produce IL-12 and iNOS, inducing a positive feedback loop that can destroy surrounding tissues. The M1-rich granulomas cannot activate HSCs to produce enough collagen deposition to protect peripheral cells from being disrupted by egg-derived antigens. Moreover, Th1 granulomas and M1 macrophages may less facilitate discharge of eggs from the host and prevent the invasion of enteric bacteria, which may cause septicemia, endotoxemia, and acute mortality. Indeed, Th1-dependent immunity produces minimal liver fibrosis, which does not bring about portal hypertension at the chronic phrase of schistosomiasis readily (7). Thus, in order to decrease mortality of schistosomiasis at both acute and chronic stage, it is indispensable to induce an immune microenvironment in liver where there is an appropriate, slight Th1-biased, M1 macrophage-dominant response, which may result in smaller granuloma formation and reduce collagen deposition, meanwhile, there are downregulated but still present M2 macrophages and Th2 response that limit egg antigens release and tissue injury (7). Herein, we explored *in vivo* how LV carrying *gra15_II_* gene (LV-*gra15_II_*)-activated liver macrophage (LV-*gra15_II_*-M) plays the inhibitory role in liver fibrosis using a schistosomiasis mouse model which has more similarity to human schistosomiasis in immune response and pathology compared with any other liver fibrosis models (7). In our previous study, we transfused murine macrophage cell line RAW 264.7 that had been activated into M1 macrophage *in vitro* by LV-*gra15_II_*, into schistosomiasis mice to induce a slight M1 macrophage-skewed immune status in liver. The result showed that these mice had significantly improved liver granulomas and fibrosis. However, the *in vivo* effect of direct delivery of LV-*gra15_II_* on attenuation of hepatic fibrotic process and its precise mechanism remains unclarified.

In the present experiments, we amplified *gra15_II_* gene from the *T. gondii* type II (PRU) strain, and constructed LV-*gra15_II_*. Then, we injected mice with LV-*gra15_II_via* tail vein. The mice were challenged with *S. japonicum* cercariae percutaneously. Eight weeks later, biochemical analyses of mice sera and pathological evaluation of mice liver were performed. We found that macrophages from the liver tissues of LV-*gra15_II_*-treated mice presented M1 cell phenotypes. We also noted that pathological process of liver fibrosis of the animals was significantly ameliorated due to re-construction of M1 macrophage-dominant immune environment by increasing matrix metalloproteinase 13 (MMP13) secretions and facilitating HSC apoptosis *in vivo*.

## Materials and Methods

### Materials

Dulbecco’s modified Eagle’s medium (DMEM) and fetal bovine serum (FBS) were purchased from Wisent (Montreal, QC, Canada). Penicillin and streptomycin were obtained from Sigma (St. Louis, MO, USA). BCA protein assay kit, SDS-polyacrylamide gel electrophoresis and 10% buffered neutral formaldehyde were purchased from Beyotime (Shanghai, China). Nitrocellulose membrane was provided by Millipore (Billerica, MA, USA). FITC-labeled anti-mouse F4/80, PerCD/cy5.5-labeled anti-mouse CD11b, PE-conjugated anti-mouse iNOS, APC-labeled anti-mouse mannose receptor (CD206), PE-Cy7-labeled anti-mouse lymphocyte antigen 6 complex (Ly6C) monoclonal antibodies, and Annexin V-FITC Apoptosis Detection Kit I were obtained from eBioscience (San Diego, CA, USA) for flow cytometry (FCM) analysis. Hematoxylin and eosin (H&E) staining kit, Masson staining kit, transforming growth factor-beta1 (TGF-β1) antibody, and flag tag antibody were purchased from Sigma (St. Louis, MO, USA). Phenylmethanesulfonyl fluoride and radio-immunoprecipitation assay (RIPA) lyses buffer were obtained from Millipore (Billerica, MA, USA). Antibodies against iNOS, Arg-1, and glyceraldehyde-3-phosphate dehydrogenase (GAPDH) were purchased from Proteintech (Chicago, IL, USA). Antibody against α-smooth muscle actin (α-SMA) was obtained from Abcam (Cambridge, MA, USA). Antibody against type I collagen (Col I) and antibody against MMP13 were manufactured by Bioworld (Minneapolis, MN, USA) for immunohistochemistry (IHC) analyses. Enzyme-linked immunosorbent assay (ELISA) kits for C-C motif chemokine ligand 2 (CCL2), NO, Col I, and MMP13 were obtained from R&D Systems (Minneapolis, MN, USA). ELISA kit for serum hyaluronic acid (HA) was purchased from CUSABIO (Wuhan, China). ELISA kit for serum amino transaminase (ALT) was acquired from Kang Lang Biological Technology Co., Ltd. (Shanghai, China). The Griess Reagent System for nitrite and hydroxyproline assay kit for hepatic hydroxyproline (HYP) was purchased from Promega Biotech Company (Madision, WI, USA) and Jiancheng Biological Engineering Research Institute (Nanjing, China), respectively. MMP13 inhibitor was purchased from MedChem Expression (Monmouth Junction, NJ, USA). Horseradish peroxide (HRP)-conjugated goat anti-rabbit IgG and the HRP-labeled goat anti-mouse were purchased from Proteintech (Chicago, IL, USA). Percoll was purchased from GE Healthcare Life Sciences (Pittsburgh, PA, USA). TRIzol was purchased from Invitrogen (Carlsbad, CA, USA). Prime Script first was purchased from TaKaRa (Beijing, China). Collagenase IV was purchased from Sigma (St. Louis, MO, USA) HSC line (JS1) was presented by Dr. Jinsheng Guo from Shanghai Zhongshan Hospital (FuDan Univ Div Digest Dis, China).

### Parasites and LV-*gra15_II_* Construction

**Figure 1:**
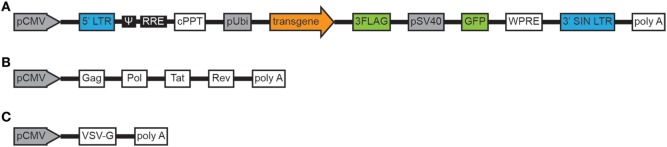
Schema of the plasmids constituting the lentiviral vector. Backbone plasmid **(A)**. Helper plasmid 1.0 **(B)**. Helper plasmid 2.0 **(C)**. Abbreviations: pCMV, cytomegalovirus promoter; LTR, long terminal repeat; ψ, packaging signal; RRE, rev responsive element; cPPT, central polypurine tract; pUbi, ubiquitin promoter; 3FLAG, three flag tags; pSV40, simian virus 40 promoter; GFP, green fluorescent protein; WPRE, woodchuck hepatitis virus posttranscriptional regulatory element; 3′SIN LTR, 3′ self-inactivating LTR, polyA, bovine growth hormone polyadenylation signal, Gag, structural proteins (Pol), replication enzymes, Tat, trans-activator; Rev, regulatory proteins; VSV-G, vesicular stomatitis virus glycoprotein.

*Toxoplasma gondii* tachyzoites from PRU cysts (type II strain) were harvested from a laboratory mouse passage at 30 days post-infection. Snails (*Oncomelania hupensis*) infected with *Schistosoma japonicum* (*S. japonicum*) were purchased from the Jiangsu Institute of Parasitic Disease Control (Wuxi, China) and were used for cercariae shedding. Amplification of gene encoding GRA15_II_ (1,500 bp, ToxoDB.org) was acquired through reverse transcription (RT)-PCR from the whole PRU strain cyst RNA. The primers were as follows: *gra15_II_*, forward 5′-CGCTCGAGAATAATTCGGTGGCTTG-3′ (the XhoI site is underlined) and reverse 5′-AGGGATCCTTCATGGAGTTACCGCTGATTG-3′ (the BamHI site is underlined). Primer synthesis and gene sequencing were performed by Sangon Biotech (Shanghai, China). Amplified gene fragment (*gra15_II_*) was directionally cloned into the pEGFP-C2 vector (BD Biosciences, Franklin Lakes, NJ, USA) with which *Escherichia coli* DH5α was transformed. The recombinant LV LV-*gra15_II_* containing three flag tags and green fluorescent protein genes was constructed by GeneChem Company (Shanghai, China) as following: lentivirus particles were produced by co-transfecting 293 T cells with three plasmids, backbone plasmid, helper plasmid 1.0, and helper plasmid 2.0, using calcium phosphate-mediated transient transfection. Engineering of self-inactivating lentiviral transfer vector by deleting the enhancer/promoter unit in the U3 region of the 3′ long terminal repeat (LTR) had minimized the risk of replication-competent lentivirus and had decreased promoter interference, which improved both performance and safety. The LV expressing *gra15_II_* was constructed by inserting *gra15_II_* cDNA into the backbone plasmid. In helper plasmid 1.0, pathogenicity genes had been eradicated, while tat gene sequence had been retained in order to enhance the tropism of LV to cells. In helper plasmid 2.0, the vesicular stomatitis virus glycoprotein (VSV-G) had taken the place of HIV-1 ENV protein, which decreased the possibility of the generation of revertant replication-competent HIV-1, increased LV stability and tropism (Figures 1A–C). Then the transfected 293 T cells were cultured in DMEM medium with 10% FBS and maintained in a 5% CO_2_ incubator at 37°C. At 48 h post transfection, culture supernatant was collected and purified with a sterilized membrane filter (0.22 μm). Subsequently, the filtered supernatant was centrifuged at 4°C, 57,500 *g* for 2 h. Then the sediment was suspended in DMEM medium, and centrifuged again at 4°C, 9,500 *g* for 5 min. The supernatant containing virus particles was collected and the titer of LV determined by p24 ELISA. The unit of titer was then converted into transduction units (TU)/ml.

### Experimental Animals and Schistosome Cercariae Challenge

Female BALB/c mice (Specific Pathogen Free, SPF), 6–8 weeks old, average weight 18–20 g, were obtained from the Changzhou Cavens Laboratory Animal Company, China (production permit number: Scxk 2011-003). The mice were housed under controlled conditions (12/12 h light/dark cycle and 22 ± 2°C temperature), and fed with standard food and pure water. All procedures were performed in strict accordance with the Chinese National Institute of Health Guide for the Care and Use of Laboratory Animals. This animal experiment was approved by the Institutional Review Board of the Institute of Biomedicine at Anhui Medical University (permit number: AMU26-093628). The mice were randomly divided into 3 groups, 24 in each: PBS group, LV-blank (LV without *gra15_II_*) group, and LV-*gra15_II_* group. All mice were challenged with 15 ± 2 cercariae through abdominal skin. The dose and frequency of LV injections were based on the pilot-experiments, two times of injection with 5 × 10^8^ TU/kg LV each. Each mouse in LV-*gra15_II_* group was injected with LV-*gra15_II_* (5 × 10^8^ TU/kg diluted in 100 μl PBS) at 7 days before cercariae challenge. Meanwhile, the mice of LV-blank group or PBS group were injected with the same dose of LV-blank or the same volume of PBS, respectively. All mice received a second injection on the day of cercariae challenge and were euthanized at the 8th week after challenge. Blood samples were collected and centrifuged for 10 min (1, 200 *g*, 4°C) for sera isolation. The liver was removed, and its left lobe was fixed with 10% neutral buffered formalin for 24 h at room temperature and embedded in paraffin for pathological and immunohistochemical examinations. The remaining liver tissues were prepared for egg counting, HYP, RNA, and protein analysis.

### Isolation of Liver Macrophages

Macrophages were isolated from mouse liver at the 8th week after challenge. First, the liver was perfused with 20 ml 0.05% type IV collagenase in Hanks’ balanced salt solution (3.5 ml/min) and removed for mincing with scissors. The shred liver tissue was digested by type IV collagenase and filtered through sterile nylon gauze (50 nm). The filtrates were washed thoroughly and isolated by 50/25% two-step Percoll density gradient centrifugation. The cells between the layers of the 25 and the 50% Percoll solution were carefully extracted.

### FCM Assay

The liver macrophages were further purified by FCM according to macrophage surface markers. In brief, all isolated macrophages were washed in PBS containing 1% FBS, then adjusted to 1 × 10^6^ cells per 100 μl PBS. The cells were subjected to FITC-labeled anti-mouse F4/80, PerCD/cy5.5-labeled anti-mouse CD11b for surface antigens staining. All macrophages were incubated with the antibodies at 4°C for 30 min and protected from light, then washed twice in PBS before detected by FCM. The liver macrophages activation status (M1 or M2) was determined through PE-labeled anti-mouse iNOS and APC-labeled anti-mouse CD206. Macrophages that showed a high expression of iNOS were further detected for Ly6C through PE-Cy7-labeled anti-mouse Ly6C. JS1 cells apoptosis was assessed through FITC/PI staining. Results of FCM were analyzed by Flow Jo software.

### Cell Culture and Co-Culture

The isolated liver macrophages were cultured in DMEM supplemented with 10–15% FBS, 2 mM l-glutamine (Gibco, Grand Island, New York, NY, USA), and 1% penicillin–streptomycin at 37°C with 5% CO_2_. The JS1 cells were maintained in the same medium as the liver macrophages except for the absence of l-glutamine. Transwell devices were used with a 0.4-μm pore size polycarbonate filter membrane, which allows small and soluble molecules but not cells to pass through. JS1 cells (1.5 × 10^5^) were seeded into lower chamber in a 12-well plate. LV-*gra15_II_*-Ms, LV-blank-containing liver macrophages (LV-blank-Ms), and liver macrophages from mice injected with PBS (PBS-Ms) (2 × 10^5^, respectively) were separately seeded into upper chamber. After 48 h co-culture, the JS1 cells were harvested for apoptosis assessment. Furthermore, the MMP13 inhibitor was added to upper chamber, and then LV-*gra15_II_*-Ms were co-cultured with JS1 cells for 72 h for Col I assay. Culture supernatants were collected after liver macrophages were cultured alone for 48 h to examine MMP13, TNF-α, CCL2, and NO.

### Hydroxyproline (HYP) and NO Detection

HYP content in liver was measured according to instruction of the hydroxyproline assay kit. The live macrophages supernatants were collected for NO measurements. The NO content reflected as the nitrite concentration was analyzed using the Griess Reagent System following the manufacturer’s instruction. The absorbance was measured at 550 nm on an ELISA reader.

### Liver Histological Examination

The liver specimens embedded in paraffin blocks were cut into 4-mm-thick sections to examine areas of the hepatic granulomas and fibrosis by H&E and Masson staining, respectively, under the manufacturer’s instructions. Bright-field images were photographed, and the areas were estimated quantitatively with computer image analysis system (Image-Pro Plus software, Media Cybernetics, Inc., Rockville, MD, USA). The same portion of hepatic tissues was weighed accurately and digested in 5% sodium hydroxide (NaOH) solution at 65°C for 1 h to count *S. japonicum* eggs.

### Western Blotting Analysis

Around 100 mg of liver tissue and the isolated liver macrophages were lysed in the ice-cold RIPA lyses buffer supplemented with protease inhibitors, and the total protein concentrations were detected by BCA protein assay kit. The proteins (20 μg) were separated on 10/6% polyacrylamide gels and electrophoretically transferred into a nitrocellulose membrane. Non-specific binding was blocked with 5% skim milk in PBS-Tween-20 (0.1%) for 2 h at room temperature. The membranes were incubated with primary antibodies to iNOS (1:1,000), Arg-1 (1:800), Flag tag (1:1,000), and GAPDH (1:2,000) at 4°C overnight, and then with HRP-conjugated secondary antibody for 1 h at room temperature. The specific signals were detected using an ECL kit; the intensity of bands in images was semi-quantitatively estimated.

### Quantitative Real-Time PCR (qRT-PCR)

RNA of the hepatic tissue and liver macrophages were extracted using the TRIzol reagent, followed by determining RNA concentration and purity by NanoDrop2000 (Thermo Scientific, Shanghai, China). RNA 1 μg was reversely transcribed to cDNA using Prime Script first Strand cDNA Synthesis Kit. The qRT-PCR was performed to examine the expression of Arg-1, α-SMA, TGF-β1, iNOS, MMP13, CCL2, TNF-α, and hepatocyte growth factor (HGF) using SYBR Premix Ex Taq kit (Vazyme, Nanjing, China) by the ABI Prism 7500 sequence detection system (Applied Biosystems, Foster City, CA, USA) following the manufacturers’ guidance. The thermal cycling condition was programmed based on the manufacturer’s instructions. GAPDH was used for normalization and as a control for the relative quantitative evaluation of the transcript abundance. Gene expression values from the qRT-PCR were analyzed using the threshold cycle (2^−ΔΔ Ct^) method. All qRT-PCR reactions were performed in technical triplicates. The forward and reverse primers are listed in Table 1.

**Table 1:** List of primers with respective sequences used for quantitative real-time PCR.

Primer	Forward	Reverse
HGF	GAGTATAGCACCATGGCCTCG	TCATCAGACACCACACCGGCACAA
CCL2	AACTCTCACTGAAGCCAGCTCT	CGTTAACTGCATCTGGCTGA
TGF-β1	CTGGATACCAACTACTGCTTCAG	TTGGTTGTAGAGGGCAAGGACCT
TNF-α	ACGGCATGGATCTCAAAGAC	GTGGGTGAGGAGCACGTAGT
iNOS	CACCTTGGAGTTCACCCAGT	ACCACTCGTACTTGGGATGC
MMP13	ACTTAACTTACAGGATTGTGA	GTGCCATCATAGATTCTGGTG
Arg-1	CTCCAAGCCAAAGTCCTTAGAG	AGGAGCTATCATTAGGGACATC
α-SMA	GGGAGCAGAACAGAGGAATG	CCAAACAAGGAGCAAAGACG
GAPDH	CAACTTTGGCATTGTGGAAGG	ACACATTGGGGGTAGGAACAC

*HGF, hepatocyte growth factor; CCL2, C-C motif chemokine ligand 2; TGF-β1, transforming growth factor beta-1; TNF-α, tumor necrosis factor; iNOS, inducible nitric synthase; MMP13, matrix metalloproteinase 13; Arg-1, arginase-1; α-SMA, α-smooth muscle actin; GAPDH, glyceraldehyde-3-phosphate dehydrogenase*.

### Immunohistochemical Analyses

The antigen retrieval was performed under high pressure in EDTA buffer. Then, the liver tissue sections were incubated with primary antibodies to immunostaining for α-SMA (1:1,000), Col I (1:250), TGF-β1 (1:300), and MMP13 (1:150) at 4°C overnight. Finally, the samples were incubated with biotinylated secondary antibodies for 45 min. Bright-field images were photographed, and the optical density in images was estimated quantitatively with computer image analysis system (Image-Pro Plus software).

### ELISA Assays

Serum HA concentration and ALT level were determined by ELISA kit according to the manufacturer’s recommendation, respectively. The live macrophages supernatants were collected for evaluation of the Col I, MMP13, TNF-α, and CCL2 by ELISA in accordance with the manufacturer’s instructions. The absorbances were measured at 450 nm on ELISA reader (Biotek, Germany).

### Statistical Analysis

The data were acquired from triplicate values representing three independent experiments with identical conditions. One-way ANOVA followed by the Bonferroni *post hoc* test was used for data analysis using SPSS *ver*. 17 (Chicago, IL, USA). Statistical significance was assessed as mean ± SD (*n* = 4 replicates for each group), two-tailed *P* < 0.05 or *P* < 0.01 was deemed statistically significant.

## Results

### LV-*gra15_II_* in the Liver and Drove Liver Macrophages to M1 Skewing

**Figure 2:**
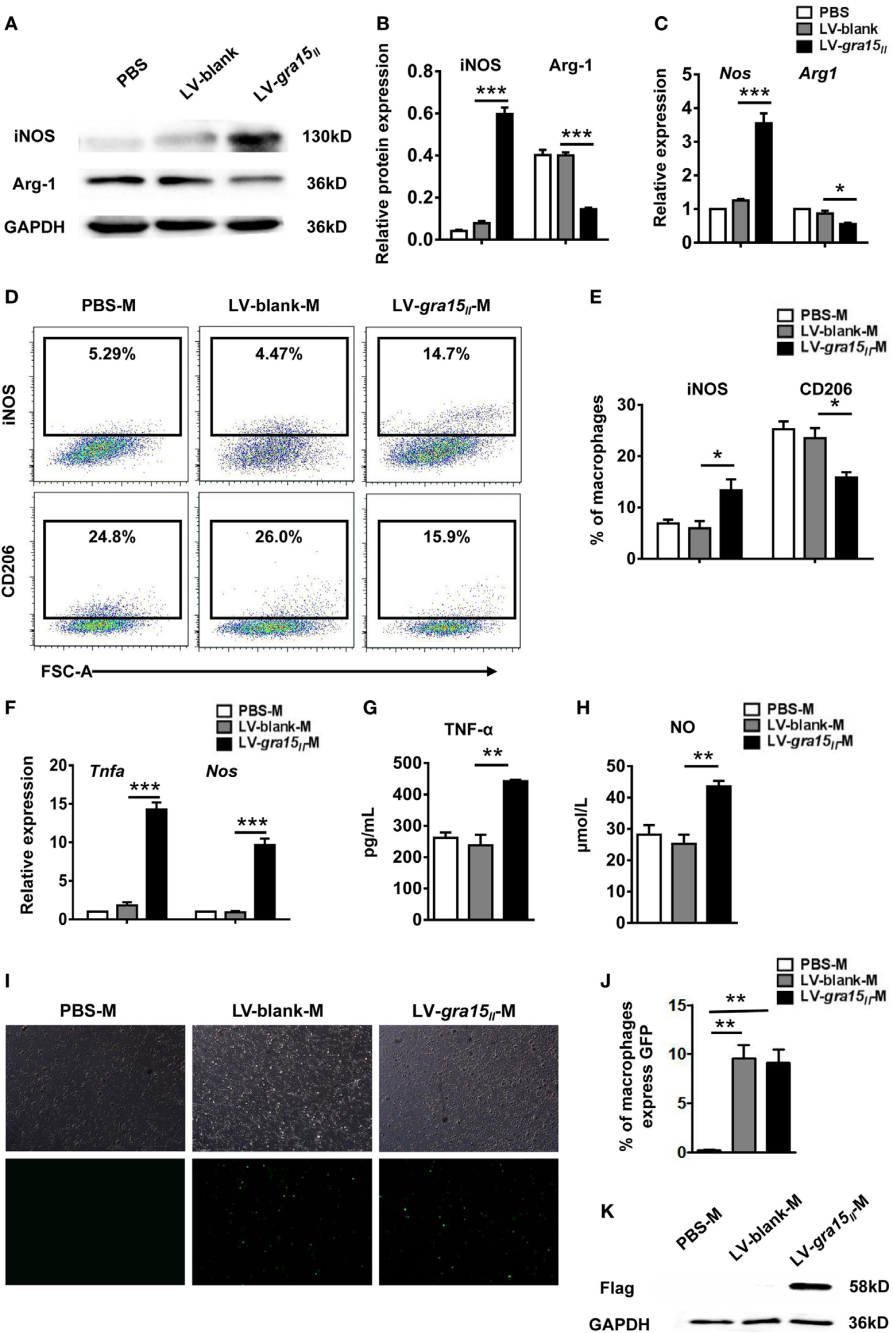
Lentiviral vector-*gra15_II_* entered to liver and induced liver macrophage into M1-like phenotype in mice. Liver protein was extracted and the production of inducible nitric oxide synthase (iNOS) and Arg-1 were detected by Western blotting **(A,B)**. Liver RNA was extracted and the expression of iNOS and Arg-1 were analyzed by quantitative real-time PCR (qRT-PCR) **(C)**. Macrophages were isolated from liver by density gradient centrifugation, and then were purified according to surface marker of F4/80 and CD11b by flow cytometry. The liver macrophage polarization state was determined by the expression of inducible nitric oxide synthase and CD206 **(D,E)**. The liver macrophages were analyzed for the expression of tumor necrosis factor-alpha (TNF-α) and iNOS by qRT-PCR **(F)**. Culture supernatants of the liver macrophages were collected and assayed for the secretion of TNF-α and nitric oxide by enzyme-linked immunosorbent assay and Griess Reagent System, respectively **(G,H)**. Green fluorescent protein in the liver macrophages were observed under fluorescence microscope **(I,J)**. The flag tag in the liver macrophages was detected by Western blotting **(K)**. Data represent mean ± SD from multi-group experiments. *p < 0.05, **p < 0.01, and ***p < 0.001 (*n* = 4 each group).

To identify whether LV-*gra15_II_* has entered the liver and induced liver macrophage activation to M1 bias, first detection of iNOS and Arg-1 protein and their mRNA expression in liver tissues was conducted by Western blotting and qRT-PCR, respectively. The results showed a significant increase of iNOS but a notable decrease of Arg-1 production in LV-*gra15_II_* group compared with LV-blank group (Figures 2A–C), suggesting a bias of liver macrophages with M1 phenotype in the liver tissues in LV-*gra15_II_* group. Subsequently, the liver macrophages were isolated by density gradient centrifugation and then purified by FCM based on cell-surface markers of F4/80 and CD11b. Macrophage activation state in liver tissues was determined by FCM according to the expression of iNOS and CD206 (Figure 2D). The data indicated that iNOS expression was significantly increased, while CD206 expression was remarkably decreased (Figure 2E) in LV-*gra15_II_* group compared with the LV-blank control. Furthermore, we harvested the liver macrophages for measurements of TNF-α and iNOS by qRT-PCR and collected the culture supernatants for detection of TNF-α and NO by ELISA and Griess Reagent System. The results revealed that TNF-α, iNOS, and NO production were markedly elevated in LV-*gra15_II_* group compared with LV-blank group (Figures 2F–H). To verify whether M1-like macrophages were polarized by GRA15_II_ protein expressed in the macrophages transduced by LV-*gra15_II_*, we examined the liver macrophages under the fluorescence microscope and found that the macrophages from both LV-*gra15_II_* and LV-blank group exhibited bright green fluorescence. The percentage of macrophages expressing GFP was significantly increased in LV-*gra15_II_*-M and LV-blank-M when compared with PBS-M control (Figures 2I,J). Western blotting detection, however, revealed that only the macrophages isolated from the liver of mice injected with LV-*gra15_II_* expressed Flag tag (Figure 2K), suggesting that the M1-like macrophages were induced by GRA15macrophages were induced protein. More interestingly, we found that the GRA15macrophages were induced expression in the liver macrophages might be sustained at least 2 months.

### LV-*gra15_II_*-M Secreted CCL2 in the Liver

**Figure 3:**
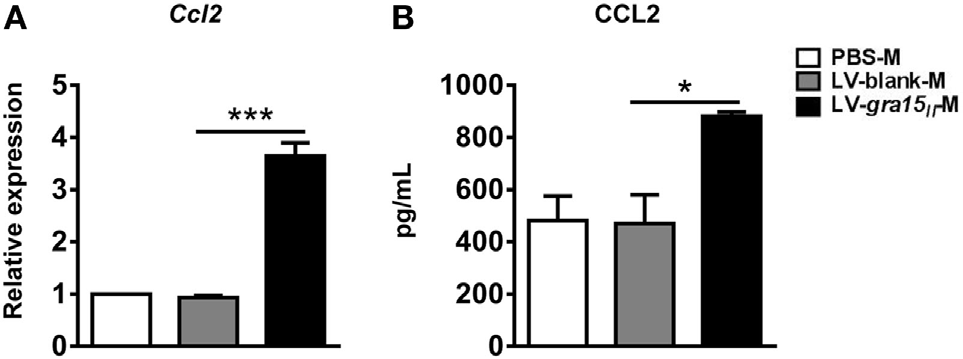
Lentiviral vector-*gra15_II_*-M increased the expression of C-C motif chemokine ligand 2 (CCL2). The liver macrophage RNA was extracted and the expression of CCL2 was analyzed by qRT-PCR **(A)**. Culture supernatants of the liver macrophages were collected and assayed for the secretion of CCL2 by enzyme-linked immunosorbent assay **(B)**. Data represent mean ± SD from multi-group experiments. *p < 0.05 and ***p < 0.001 (*n* = 4 each group).

C-C motif chemokine ligand 2 is a profibrogenic chemokine, which can recruit monocytes/natural killer (NK) cells into liver tissue. These cells are associated with HSC apoptosis and liver fibrosis (1). The expression of CCL2 mRNA in the LV-*gra15_II_*-M or CCL2 protein in the culture supernatants was greatly increased when compared with those in LV-blank-M control (Figures 3A,B), suggesting that LV-*gra15_II_*-M was involved in anti-fibrosis activity through recruiting endogenous macrophages and NK cells by secreting CCL2 (1).

### LV-*gra15_II_* Delivery Alleviated Hepatic Granulomas and Fibrosis

**Figure 4:**
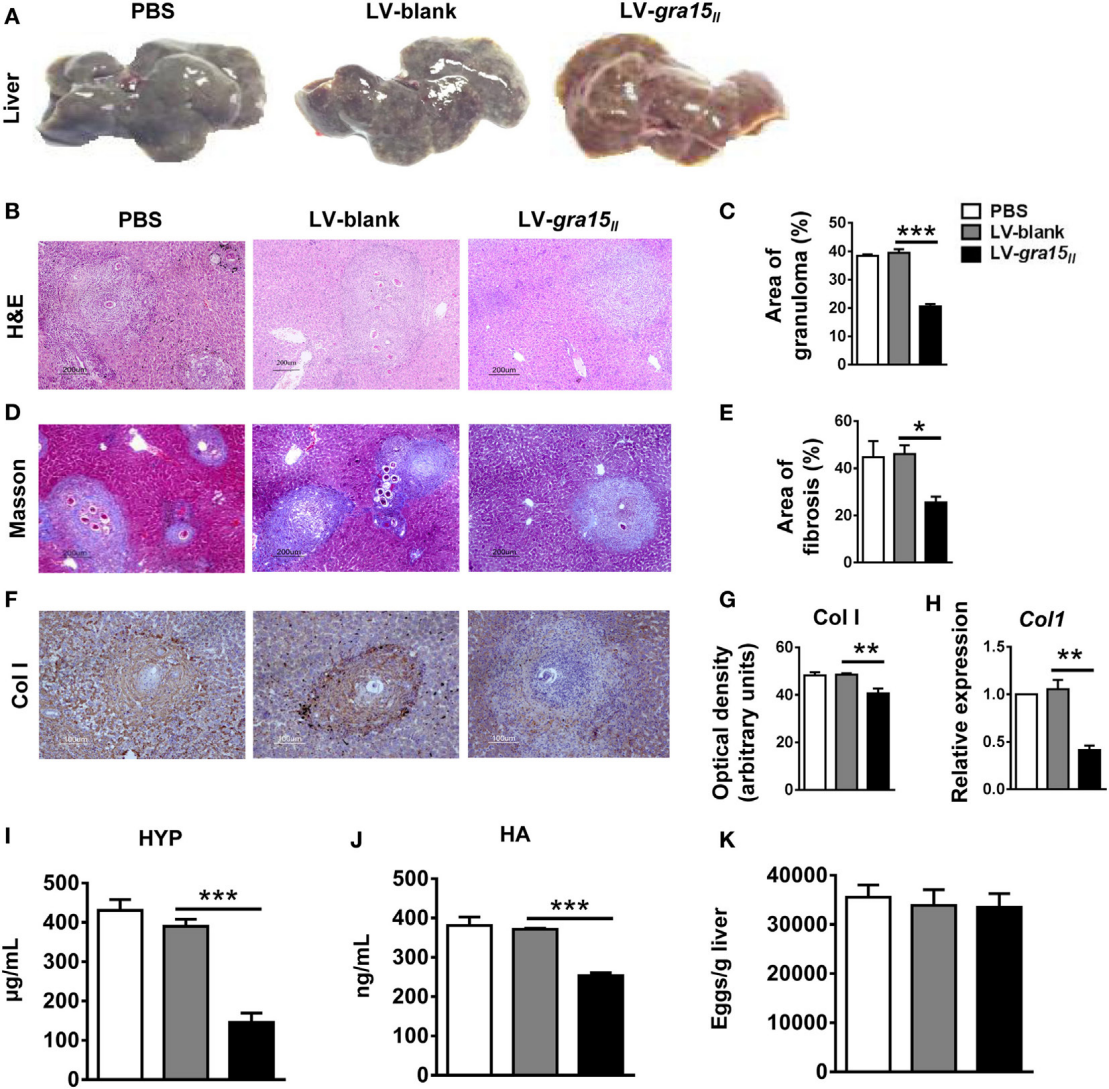
Lentiviral vector-*gra15_II_* delivery ameliorated hepatic granulomas and fibrosis in schistosomiasis mice. Mice were killed under euthanasia at 8 weeks post-infection, and then the blood and the liver were collected. The appearances of liver were exhibited **(A)**. Liver tissue section was stained with hematoxylin and eosin and the granulomas area was showed with quantitative analysis **(B,C)**. Liver tissue section was stained with Masson and the fibrosis area was showed with quantitative analysis **(D,E)**. Col Ι protein level in liver was evaluated by IHC with quantitative analysis of optical density **(F,G)** and Col Ι gene expression in liver was analyzed by quantitative real-time PCR **(H)**. The HYP content in liver was checked by hydroxyproline assay kit **(I)**. Sera hyaluronic acid level was assayed by enzyme-linked immunosorbent assay **(J)**. Eggs in liver tissue were counted under light microscope **(K)**. Data represent mean ± SD from multi-group experiments. *p < 0.05, **p < 0.01, and ***p < 0.001 (*n* = 4 each group).

To validate the effect of M1 activation on liver fibrosis, we evaluated the liver pathology in the following experiments. The appearance, color, and hardness of liver were almost normal with just several granulomas nodules on the liver surface in LV-*gra15_II_* group (Figures 4A). Liver tissue H&E (Figures 4B,C) and Masson (Figures 4D,E) staining demonstrated that the areas of hepatic granulomas and fibrosis were both significantly reduced in LV-*gra15_II_* group compared with LV-blank group. Both immunohistochemistry staining and qRT-PCR analysis showed that the expression of liver Col I was downregulated in LV-*gra15_II_* group (Figures 4F–H). Moreover, the content of liver HYP and the level of sera HA were decreased in LV-*gra15_II_* group compared with LV-blank group (Figures 4I,J). Interestingly, the number of eggs counted under a light microscope showed no significant difference among three groups (Figure 4K). These results suggested that M1-dominant immunity induced by GRA15_II_ played an inhibitory role in the process of hepatic granulomas and fibrosis although it had no significant impact on the deposition of eggs in the liver tissues. Additionally, we noted the normal vitality of mice in LV-*gra15_II_* group compared with the control animals.

### LV-*gra15_II_* Delivery Deactivated HSC *via* Inhibiting TGF-β1 Expression

**Figure 5:**
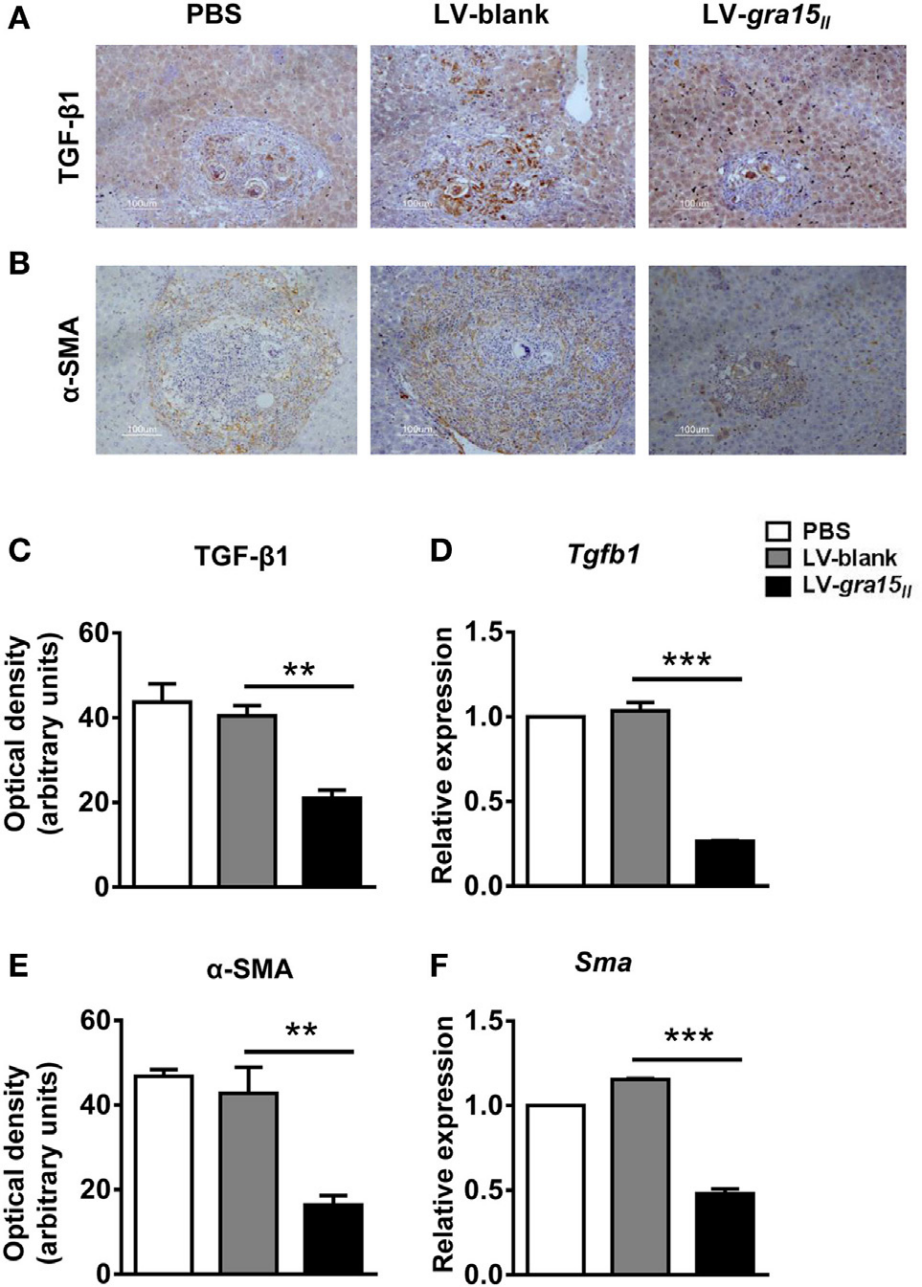
Lentiviral vector-*gra15_II_*-M delivery decreased hepatic stellate cell (HSC) activation by the reduced expression of transforming growth factor-beta1 (TGF-β1). TGF-β1 protein level in liver was evaluated by IHC with quantitative analysis of optical density **(A,C)**; the protein level of α-SMA in liver was evaluated by IHC with quantitative analysis of optical density **(B,E)**. Liver RNA was extracted and the expression of TGF-β1 and α-SMA were analyzed by quantitative real-time PCR **(D,F)**. Data represent mean ± SD from multi-group experiments. **p < 0.01 and ***p < 0.001 (*n* = 4 each group).

To clarify the mechanism of LV-*gra15_II_* delivery ameliorating hepatic granulomas and fibrosis, we compared the production of TGF-β1 in different groups. As expected, both TGF-β1 protein and mRNA expression in the liver tissues were significantly reduced in LV-*gra15_II_* group compared with LV-blank group evaluated by IHC staining and qRT-PCR, respectively (Figures 5A,C,D). Simultaneously, α-SMA protein (a marker of HSC activation) and mRNA expression, which were analyzed by IHC staining and qRT-PCR, respectively, notably declined in LV-*gra15_II_* group compared with LV-blank group (Figures 5B,E,F). The results indicated that the liver tissues of mice following LV-*gra15_II_* treatments produced less TGF-β1 which is responsible for activation of HSCs.

### LV-*gra15_II_*-M Promoted Col I Degradation *via* Secreting MMP13

**Figure 6:**
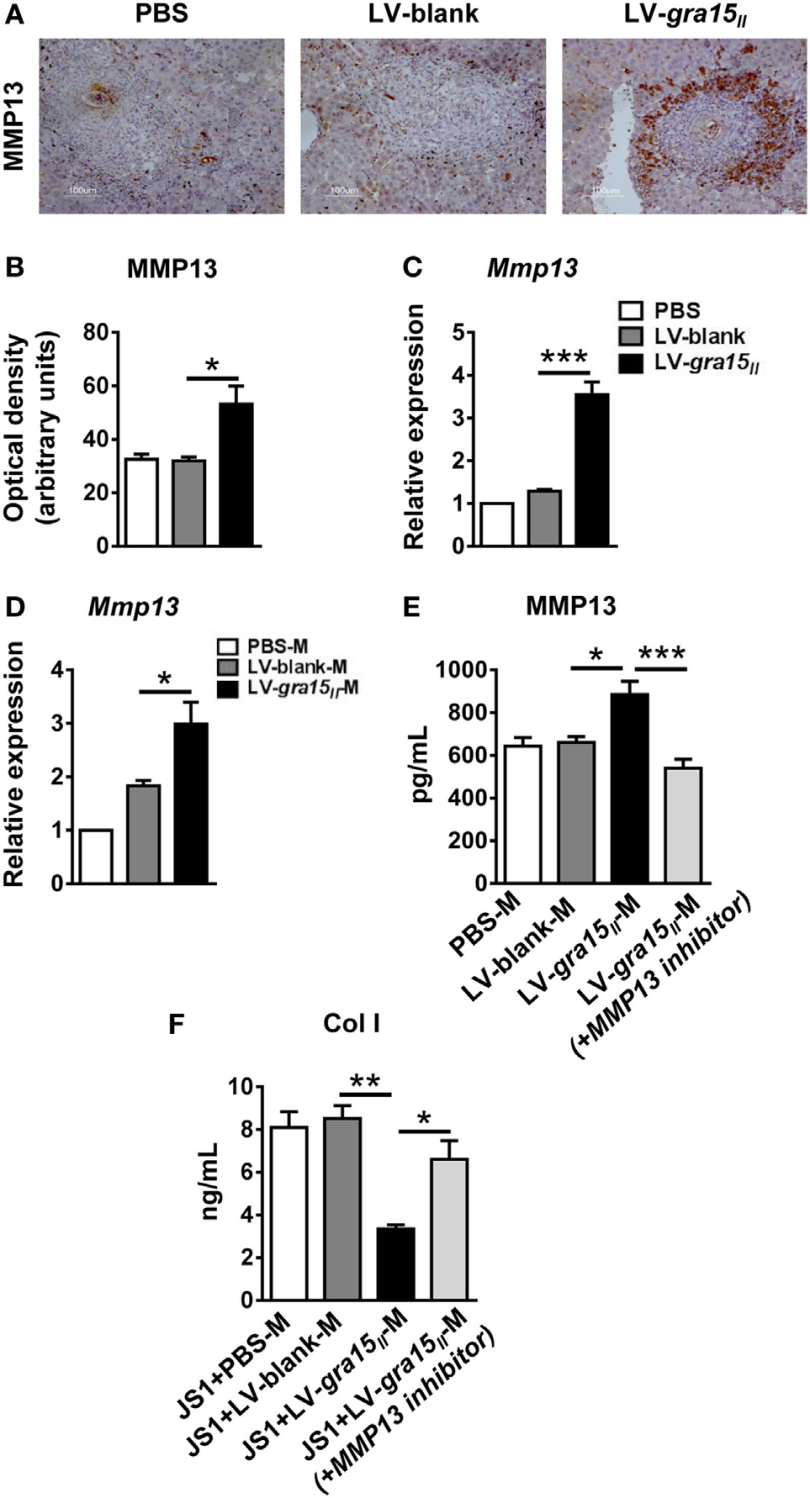
Matrix metalloproteinase 13 (MMP13) secreted from lentiviral vector-*gra15_II_*-M was an important factor which degraded Col I. The protein level of MMP13 in liver was evaluated by IHC with quantitative analysis of optical density **(A,B)**. Liver RNA was extracted and the expression of MMP13 was analyzed by quantitative real-time PCR (qRT-PCR) **(C)**. The liver macrophage RNA was extracted and the expression of MMP13 was analyzed by qRT-PCR **(D)**. Liver macrophages culture supernatants with or without MMP13 inhibitor were collected and assayed for the secretion of MMP13 by enzyme-linked immunosorbent assay (ELISA) **(E)**. Liver macrophages and JS1 cells supernatants in co-culture system with or without MMP13 inhibitor were collected and assayed for the secretion of Col Ι by ELISA **(F)**. Data represent mean ± SD from multi-group experiments. *p < 0.05, **p < 0.01, and ***p < 0.001 (*n* = 4 each group).

To further explore the mechanism by which the LV-*gra15_II_*-M alleviated hepatic granulomas and fibrosis, we investigated the MMP13 expression both *in vitro* and *in vivo*. First, we detected MMP13 protein by IHC staining and mRNA expression by qRT-PCR in the liver tissues. We found that expression of MMP13 in protein and mRNA levels was synchronously increased in LV-*gra15_II_* group compared with LV-blank group (Figures 6A–C). Moreover, we extracted the liver macrophage RNA and collected the liver macrophage culture supernatant with or without MMP13 inhibitor. Subsequently, we analyzed the expression of MMP13 in liver macrophage by qRT-PCR and detected the secretion of MMP13 in liver macrophages culture supernatant by ELISA, respectively. The results showed that the production of MMP13 was strikingly enhanced in LV-*gra15_II_*-M-treated animals compared with LV-blank-M control (Figures 6D,E). Finally, we collected the liver macrophages and JS1 cells co-culture supernatants in the co-culture system with or without MMP13 inhibitor to measure the secretion of Col I from JS1 cells by ELISA, the results showed that LV-*gra15_II_*-M significantly dampened the production of Col I compared with LV-blank-M (Figure 6F). These data strongly suggested that LV-*gra15_II_*-M involves degradation of Col I due to its facilitation to MMP13 secretion.

### LV-*gra15_II_*-M Contributed to the Apoptosis of JS1 Cells *In Vitro*

**Figure 7:**
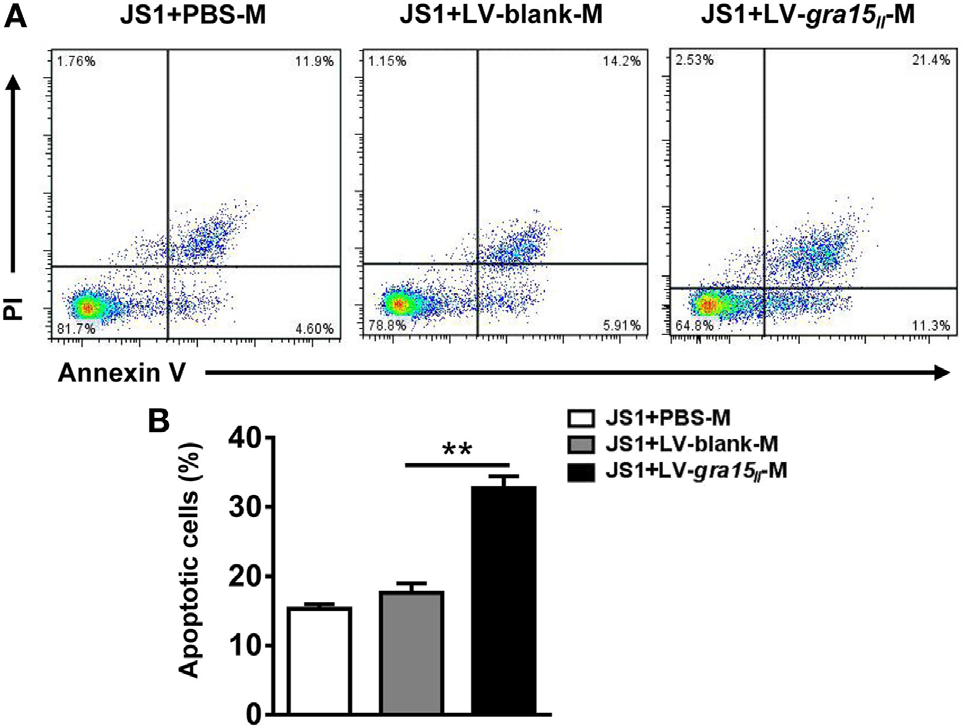
Lentiviral vector-*gra15_II_*-M promoted hepatic stellate cell apoptosis. Liver macrophages were co-cultured with JS1 cells for 48 h in transwell, and then JS1 cells were collected and assessed for apoptosis by FCM **(A,B)**. Data represent mean ± SD from multi-group experiments. **p < 0.01 (*n* = 4 each group).

There is now a clear evidence that HSC apoptosis is one of the main mechanisms of regression of fibrosis (25). We herein researched the potential role of the LV-*gra15_II_*-M in inducing HSC apoptosis *in vitro*. Liver macrophages were co-cultured with JS1 cells for 48 h in transwell device, and then JS1 cells were collected for assessment of apoptosis by FCM. The result displayed that LV-*gra15_II_*-M distinctly induced both early and late apoptosis compared with LV-blank-M (Figures 7A,B).

### LV-*gra15_II_*-M Prominently Expressed HGF and Ly6C

**Figure 8:**
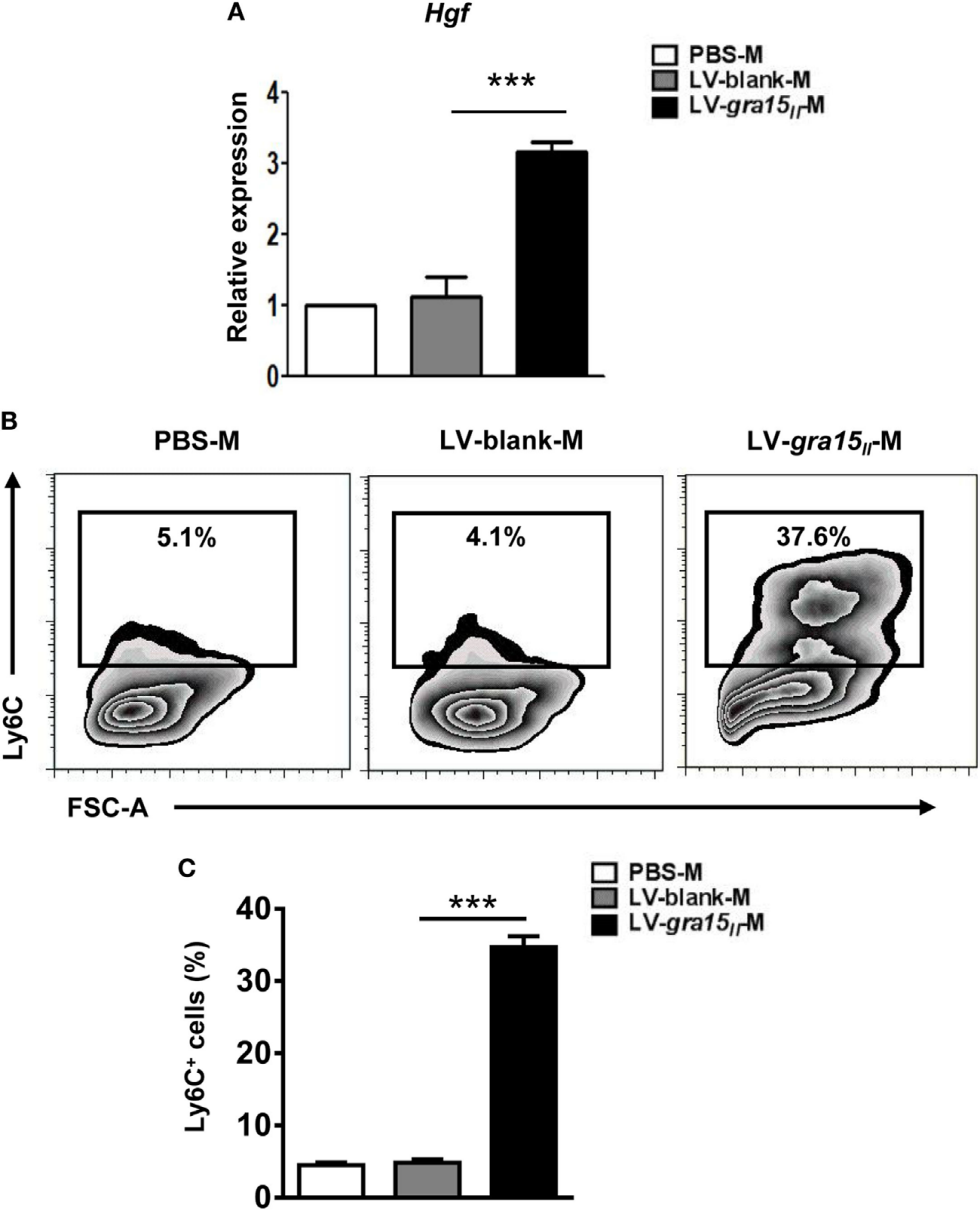
Lentiviral vector (LV)-*gra15_II_*-M had high expression of hepatocyte growth factor (HGF) and lymphocyte antigen 6 complex (Ly6C). The liver macrophages were isolated, purified, and collected from mice. The liver macrophage RNA was extracted to analyze the expression of HGF by quantitative real-time PCR **(A)**. The expression of Ly6C on the LV-*gra15_II_*-M surface was detected by FCM **(B,C)**. Data represent mean ± SD from multi-group experiments. ***p < 0.001 (*n* = 4 each group).

In order to ascertain whether the LV-*gra15_II_*-M can protect hepatocytes from immune injury, we extracted the liver macrophage RNA to analyze HGF level by qRT-PCR. The detection revealed a high expression of HGF in LV-*gra15_II_*-M compared with LV-blank-M (Figure 8A). Serum ALT level assayed by ELISA had no significant change in LV-*gra15_II_* group compared with the other groups (data not shown), suggesting no hepatocytes damage by Th1/M1-dominant immune microenvironment. Meanwhile, the expression of Ly6C detected by FCM in the LV-*gra15_II_*-M was higher than that in LV-blank-M (Figures 8B,C), but no significant difference was noted between PBS and LV-blank groups in all of the experiments (dates not shown).

## Discussion

The currently used HIV-1-based LVs are third-generation vectors with significant improvements in the safety and efficiency. Non-essential viral genes have been removed from the plasmids, so the self-inactivating LVs have reduced the risk of reconstituting pathogenic parental HIV-1 virus and recombining replication-competent vector with less possibility of insertional mutagenesis (5, 6, 26). Additionally, no anti-vector immunity has been detected against the LVs, allowing the use of a multiple injection approach (26). The LVs are generated as the VSV-G pseudotypes to allow for production of highly infectious virus with a broad tropism for target cell transduction (26). Finally, the use of a cytomegalovirus/LTR hybrid construct has enhanced vector production efficiency (5). Domenico et al. (27) showed that injection of a LV carrying targeting cDNA into the tail vein of mice resulted in expression of the transgene in several murine tissues, with the highest level reached in the liver and spleen. Brown et al. (28) reported that LVs efficiently transduced into liver macrophages and other nonparenchymal cells compared with hepatocytes, because liver macrophage is among the first cell to be exposed to the vector, and may be the most efficient at uptaking vector particles. In the present work, we used the LVs as a vehicle to explore the mechanism by which liver M1 macrophages activated by LV-*gra15_II_* ameliorated hepatic fibrosis of schistosomiasis mice.

Lentiviral vectors are promising tools for the genetic modification of cells in biomedical research and gene therapy (3). However, the risk such as immunogenicity of vector particles needs to be taken into account. Generally, immune response induced by the viral particles (e.g., structural proteins and envelopes) is usually low or absent (5), but induced by the transgene product can be a major concern for gene therapy (3). Although the systemic delivery of LVs may lead to an immune response against the transgene product or a cytotoxic T-cell response and subsequent elimination of transduced hepatocytes (29, 30), in our studies, using the serum ALT level assay, we found no immune damage to hepatocytes, which may partially be attributed to the protection of HGF secreted by LV-*gra15II*-M.

Fibrosis caused by diverse etiologies is closely related with Th2-biased immunity, which usually coexists with M2 macrophage-predominant response induced by Th2 cytokines in tissue microenvironment (31, 32). Imbalance in production and degradation of collagen, associated with Th2- and M2 macrophage-prone immune response, promotes excessive extracellular matrix (ECM) deposition. For example, schistosomiasis hepatic fibrosis is the result of M2 macrophage-rich egg granulomas and Th2-biased response (31). So, a promising strategy against schistosomiasis hepatic fibrosis is to increase M1 macrophage population or Th1 response in liver tissue to coordinate Th1/M1 and Th2/M2 balance (31, 32). In our previous study, we infused M1 macrophages activated *in vitro* by LV-*gra15_II_* into schistosomiasis mice to induce a M1-dominant response in liver, which significantly ameliorated hepatic granuloma formation and fibrosis process (33).

In the present study, we focused at the liver macrophage as one of the primary cellular components in schistosomiasis hepatic granulomas, to mitigate hepatic fibrosis (7, 12, 13, 34). The liver macrophages were induced to M1 macrophages *in vivo* by LV-*gra15_II_* delivery to schistosomiasis mice, and then hepatic granulomas and fibrosis were evaluated and the effect of LV-*gra15_II_*-M on JS1 cells apoptosis and collagen production was observed in cell co-culture system for the potential mechanism of LV-*gra15_II_* treatment against fibrosis. Our previous study indicated that, *in vitro* activated M1 macrophages were transfused into mice at 3-, 4-, 5-, or 6-week post-infection with schistosome cercariae, but not played any role in either the pathology of liver tissue or the production of fibrosis-related factors, suggesting that it is probably indispensable to pre-built a M1-biased immune microenvironment in the liver tissues before *S. japonicum* infection for reduction of hepatic fibrosis (33).

Our study confirmed that LV-*gra15_II_* not only migrated liver macrophages but also continuously drove the expression of GRA15_II_ protein for at least 2 months, which persistently polarized liver macrophages toward M1-like phenotype with increased iNOS, TNF-α, CCL2, and decreased Arg-1, CD206 expression in the liver or LV-*gra15_II_*-M. It has been known that CCL2, as a member of the monocyte chemotactic protein-1, mediates monocytes migration from blood flow into inflammatory tissue, where these monocytes are differentiated into M1 or M2 macrophages in local microenvironment (35, 36). Moreover, our pervious study showed that macrophages polarized by LV-*gra15_II_* secreted a high level of IL-12, which may drive Th1 response in inflammatory tissues (31, 33). As observed in our finding, activated M1 liver macrophages might be derived from both LV-*gra15_II_*-M and recruited monocytes activated by Th1 cytokines in liver local microenvironment. Also, M1 macrophages promote Th1 to recruit into inflammatory tissues by secreting chemokines CXCL9-11 (31). Here we have successfully created a Th1/M1 macrophage-biased immune microenvironment in schistosomiasis fibrotic liver by LV-*gra15_II_* delivery.

Immune deviation studies in schistosomiasis mice revealed that a Th1 response, which was switched from a Th2 response by IL-12, provided protection against fibrosis (37). Similar results were also identified in the CCl4-induced liver fibrosis model (38). Thus we evaluated the pathology of the liver tissues to verify the effect of M1-predominant immune response on hepatic fibrosis. In LV-*gra15_II_* group, the liver was almost similar to normal liver in appearance, color, and stiffness, except for a few granulomas nodules on the liver surface. Moreover, the areas of granulomas and fibrosis, the content of HYP and Col I in the liver, and the level of HA in the sera decreased significantly without eggs load changed. These results demonstrated that M1-dominant immunity induced by LV-*gra15_II_* had no significant impact on the egg production of the helminth but had capacity to alleviate hepatic granulomas and fibrosis.

Macrophages and HSCs are the main cell types involved in the pathogenesis of hepatic granulomas (39). M2 macrophages produce TGF-β1 that directly prompts HSCs differentiation into fibrogenic-SMA positive myofibroblasts (activated HSCs) through TGF-β1-Smad2/3 signaling (40, 41). So, in the liver tissues with M1-dominant environment, numerous HSCs were inactivated owing to the decrease of TGF-β1. Consequently, collagen deposition was reduced.

Matrix metalloproteinase 13 is the primary collagenase which degrades complex ECM components and remodels fibrillar collagen, especially Col I in rodents (42, 43). Macrophages located almost exclusively in areas of liver tissue scarring are a major source of MMP13 and can resolve liver fibrosis both directly by production of MMP13 and indirectly by secretion of cytokines, such as TNF-α and IL-1, which regulate MMP13 production by other cells (42). In our study, both immunohistochemical staining and qRT-PCR showed that MMP13 expression was noticeably enhanced in the liver after LV-*gra15_II_* treatment compared with control groups. Further experiments verified that liver macrophages in LV-*gra15_II_* group expressed more MMP13 than other groups. Moreover, when liver macrophages with high secretion of MMP13 were co-cultured with JS1 cells, the content of Col I secreted from JS1 cells was reduced distinctly. Specially, the reduction of Col I was partially blocked by MMP13 inhibitor, suggesting a key factor of MMP13 for Col I degradation. Therefore, M1 macrophage-biased immunity might be one of the main mechanisms of liver fibrogenolysis due to Col I degradation by M1 macrophage-generated MMP13.

In addition, we noted that JS1 cells apoptosis increased obviously when JS1 cells and LV-*gra15_II_*-Ms were co-cultured in transwell device. During fibrogenesis, the expression of TNF-related apoptosis-induced ligand (TRAIL) receptor is upregulated only on activated HSC. Therefore, macrophages and NK cells, which secrete TRAIL, can particularly induce activated HSC apoptosis (1, 44). Ma et al. reported that CCL2, derived from M1 macrophages, not only recruits NK cells from the blood stream into fibrotic liver, but also activates NK cells (1). Therefore, LV-*gra15_II_*-M may induce HSC apoptosis by secreting CCL2, which drives NK cells to produce TRAIL. However, unlike TRAIL receptor, death receptor (Fas receptor) is expressed both on HSCs and hepatocytes. So, the activation of the Fas receptor-mediated pathway may result in apoptosis of both cell types (25). HGF plays a crucial role in protecting hepatocyte from apoptosis (45). In the present study, HGF gene transcript in LV-*gra15_II_*-M was determined by qRT-PCR and the result displayed a high expression of HGF, which implied that LV-*gra15_II_*-M provided protection through HGF for hepatocytes against damage caused by apoptosis during liver fibrolysis. This may also help for explanation for the normal level of serum ALT and the vitality of mice in LV-*gra15_II_* treatment in schistosomiasis mice. Moreover, a growing body of evidence have demonstrated that interferon (INF)-γ and NO, which are derived from M1 macrophages, are also capable of mediating HSC apoptosis (1, 10, 44, 46).

Intrahepatic macrophages with Ly6C high expression, derived from recruitment of circulating Ly6C^high^ monocytes, are critical for fibrogenesis (47). Ramachandran et al. used a CCl4-induced model of reversible murine hepatic fibrosis to identify "restorative macrophage" as a specific macrophage population responsible for fibrosis resolution. This macrophage is derived from recruited Ly6C^high^ monocyte and has a phenotypic switch mediated by the ingestion of cellular debris in the fibrotic liver, which is different from the M1/M2 macrophage paradigm, with increased production of MMPs and decreased expression of Ly6C (48). Yet, our study manifested that LV-*gra15_II_*-M had a high expression of Ly6C with raised secretion of MMP13, increased apoptosis of HSC, and depressed secretion of TGF-β1, suggesting that expression intensity of Ly6C on surface of macrophage may be not crucial for M1 macrophages to suppress fibrosis, but production of MMPs, apoptosis of HSCs, and reduction of profibrogenic signals play key roles in fibrogenolysis. In addition, LV-*gra15_II_*-M may relieve the extent of fibrosis through reducing the production of proline and polyamine because of the enhanced expression of iNOS in the LV-*gra15_II_*-M. Perhaps, LV-*gra15_II_*-M represents a new macrophage subset, which is only activated by just LV-*gra15_II_* in just Schistosomiasis japonica fibrotic liver. In different immune microenvironments, macrophages employ distinct phenotypes and even switch between different phenotypes in response to the diverse stimulating signals to which they are exposed, and exert distinct effects on fibrosis formation, limitation, or dissolution according to their different activation status (13–15). Further exploration of the nature, phenotype, activation, gene expression, cytokine and chemokine production, metabolism, and function of LV-*gra15_II_*-M would be needed in the future, which will enrich our knowledge of macrophage phenotype activation and its function.

**Figure 9:**
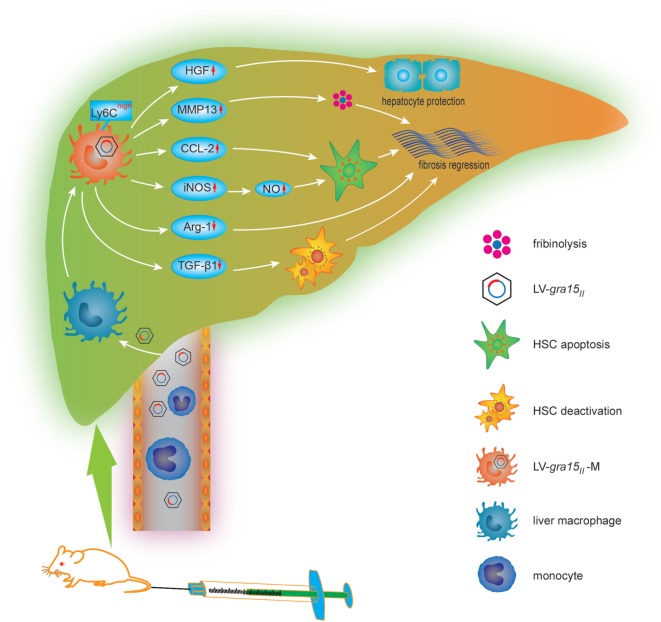
The cellular and molecular mechanisms by which lentiviral vector (LV)-*gra15_II_* delivery mitigates schistosomiasis liver fibrosis. LV-*gra15_II_* infects macrophage in fibrotic liver after being injected *via* schistosomiasis mice tail vein and transduces liver macrophage into LV-*gra15_II_*-M which presents a M1 macrophage-like phenotype. The LV-*gra15_II_*-M with a high expression of lymphocyte antigen 6 complex (Ly6C) increases the production of matrix metalloproteinase 13 (MMP13), C-C motif chemokine ligand 2 (CCL2), inducible nitric oxide synthase (iNOS), nitric oxide (NO), and decreases the generation of Arg-1, transforming growth factor-beta1, which results in fibrosis mitigation through dissolving collagen protein, inducing HSC apoptosis or deactivation and reducing fibrogenesis. Moreover, the LV-*gra15_II_*-M protects hepatocytes against apoptosis through secreting hepatocyte growth factor.

In conclusion, we studied, for the first time, the cellular and molecular mechanisms of LV-*gra15_II_*-mediated mitigation for schistosomiasis liver fibrosis (Figure 9). We hypothesized that LV-*gra15_II_* delivery might ameliorate liver fibrosis. To test this, we injected the LV-*gra15_II_* into mice followed by *S. japonicum* infection. Our studies showed that the liver macrophages in mice with schistosomiasis were induced to a special M1 macrophage phenotype with a high expression of Ly6C. As a result, hepatic granulomas and fibrosis were significantly ameliorated by the LV-*gra15_II_*-induced Th1/M1-prone immune response with the vitality of mice not affected. Furthermore, LV-*gra15_II_*-M greatly reduced the secretion of TGF-β1, increased the expression of MMP13, and promoted the apoptosis of HSC without prominent injury of hepatocytes in experimental mouse liver fibrosis. Our investigation also suggests that a slightly biased Th1/M1 macrophage response in the liver to rebalance *S. japonicum*-triggered immunity may minimize granulomas formation and alleviate fibrotic process while simultaneously protect the hepatocytes and host from damage.

## Ethics Statement

All procedures were performed in strict accordance with the Chinese National Institute of Health Guide for the Care and Use of Laboratory Animals. This animal experiment was approved by the Institutional Review Board of the Institute of Biomedicine at Anhui Medical University (permit number: AMU26-093628).

## Author Contributions

LL, JS, and DC conceived and designed the trial, and JS critically revised the manuscript. LL, MJ, QT, and other authors performed the experiments and the statistical analysis. LL and DC wrote the manuscript. All authors have read and approved the final manuscript.

## Conflict of Interest Statement

The authors declare that the research was conducted in the absence of any commercial or financial relationships that could be construed as a potential conflict of interest.
